# Replicability in Brain Imaging

**DOI:** 10.3390/brainsci12030397

**Published:** 2022-03-16

**Authors:** Robert E. Kelly, Matthew J. Hoptman

**Affiliations:** 1 Department of Psychiatry, Weill Cornell Medicine, White Plains, NY 10605, USA; 2 Clinical Research Division, Nathan S. Kline Institute for Psychiatric Research, Orangeburg, NY 10962, USA; matthew.hoptman@nki.rfmh.org; 3 Department of Psychiatry, New York University Grossman School of Medicine, New York, NY 10016, USA

In the early 2010s, the “replication crisis” and synonymous terms (“replicability crisis” and “reproducibility crisis”) were coined to describe growing concerns regarding published research results too often not being replicable, potentially undermining scientific progress [1]. Many psychologists have studied this problem, contributing groundbreaking work resulting in numerous articles and several Special Issues in journals, with titles such as “Replicability in Psychological Science: A Crisis of Confidence?”, “Reliability and replication in cognitive and affective neuroscience research”, “Replications of Important Results in Social Psychology”, “Building a cumulative psychological science”, and “The replication crisis: Implications for linguistics” [1,2,3,4,5]. Researchers in the field of brain imaging, which often dovetails with psychology, have also published numerous works on the subject, with brain imaging organizations having become staunch supporters of efforts to address the problem, such organizations including the Stanford Center for Reproducible Neuroscience and the Organization for Human Brain Mapping (OHBM), the latter having created an annual award for the best replication study [6], regularly featuring informative events concerning the replication crisis and Open Science at its annual meetings [3,7]. The purpose of the *Brain Sciences* Special Issue “The Brain Imaging Replication Crisis” is to provide a forum for discussions concerning this replication crisis in light of the special challenges posed by brain imaging.

In John Ioannidis’ widely cited article entitled “Why most published research findings are false”, he convincingly argues that most published findings are indeed false, with relatively few exceptions [8,9,10]. He supports this claim using Bayes’ theorem and some reasonable assumptions concerning published research findings. It follows from Bayes’ theorem that when a hypothesis test is positive, the likelihood that this study finding is true (PPV, positive predictive value) depends on three variables: the α-level for statistical significance (where *α* is the probability of a positive test, given that the hypothesis is false), the power of the study (1 − *β*, where *β* is the probability of a negative test, given that the hypothesis is true), and the odds that the hypothesis is true (*R*, the ratio of the probability that the hypothesis is true to the probability that the hypothesis is false). This relationship is expressed with the equation PPV = *R*(1 − *β*)/[*α* + *R*(1 − *β*)]. From this equation, it follows that any hypothesis will likely be false, even after a positive test, when *R* < *α*. This situation applies to fields where tested hypotheses are seldom true, which could in part explain the low replication rates observed in cancer studies [11,12]. It also follows that when the study power is equal to *α*, the probability that the hypothesis is true remains the same as it was before the test. Thus, inadequately powered studies lack the capacity to advance our confidence in the tested hypotheses. The PPV can also be reduced by sources of bias that elevate the actual value of *α* above its nominal value, for example, when publication bias [13,14] causes only positive studies to be published for a given hypothesis. When published *p*-values are not corrected for multiple comparisons involving negative studies, actual *p*-values become much higher than the published ones.

Academic incentives regarding the publication of “interesting” findings in high-impact journals can further bias research towards the production of spurious, false–positive findings through multiple mechanisms [15]. Simmons et al. [16] demonstrated with computer simulations how four common variations in research methods and data analyses allowed the inflation of actual *p*-values via so-called p-hacking [14,17], from 0.05 to 0.61. Researchers incentivized to find their anticipated results might be biased towards choosing methods that yield those results [18]. In the same vein, methodological errors [19] might be found less frequently when they support the anticipated results. Additionally, after seeing the results of a study, researchers might be inclined to reconsider their original hypotheses to match the observed data, so-called HARKing (hypothesizing after the results are known) [20].

To counteract these deleterious academic incentives, Serra-Garcia and Gneezy [21] proposed disincentives for the publication of nonreplicable research findings. A problem with this approach is that it can take years and considerable research resources to identify such findings. Another problem is that the replicability of findings is not necessarily a good measure of study quality. High-quality studies have the capacity to sift out replicable from irreplicable hypotheses, for example, in confirmatory studies to provide a higher margin of certainty for hypotheses already considered likely to be true, and in exploratory studies to identify promising candidates for further research. Obviously, some such candidate hypotheses will not prove replicable. Conversely, a positive study of low quality, with no capacity to separate true from false hypotheses, could prove replicable if the tested hypothesis happened to be true.

Determining which hypotheses are replicable can be especially challenging in the field of brain imaging, with many experiments lacking the power to find the sought-after differences in neural activity due to limitations in the reliability of measures combined with cost considerations limiting sample sizes [22,23,24,25,26,27,28,29,30]. Nonetheless, the countless pipelines from available methods of analysis can provide the needed *p*-value to support practically any hypothesis [31,32]. HARKing also reliably yields positive findings, which can seem confirmatory. For example, if using functional connectivity (FC) to study brain differences between two groups that differ clinically in some way, one recipe for “success” is the following: (1) divide the brain into ~100 regions and find the FC between each pair of regions, yielding ~500 such pairs whose FC differs significantly between the two groups, with α = 0.05; (2) select a pair of such brain regions that happens to correspond to existing findings in the literature related to the studied clinical group differences; (3) write the paper as if the selected pair had been the only pair of interest, based on the literature search, thereby giving the appearance that the study is a confirmation of an expected finding.

What can improve the replicability of research results? Theoretical considerations can help to sift out likely from unlikely hypotheses even before testing begins [33]. Judicious study design can improve power. Perhaps the most efficient means of improving replicability are those that address the inflation of *p*-values. The preregistration of study hypotheses and methods [3,7] can prevent p-hacking and HARKing, provided that methods are specified in enough detail to eliminate flexibility in the data collection and analysis. A detailed specification of methods in published articles allows other researchers to reproduce published studies and to double-check the authors’ work if study data and software are also available. Many organizations now provide tools to facilitate such a preregistration of studies and storage of data and software. The Center for Open Science [34,35], for example, is a well-funded, nonprofit organization that provides these services at little to no cost to researchers.

We welcome the submission of papers contributing further ideas for how to address the replication crisis, including replication studies or papers describing refinements of brain imaging methods to improve study power. Additionally welcome are examples of excellent study quality involving (1) preregistration with detailed methods allowing an unambiguous study reproduction and (2) availability of data and software, if feasible. Please feel free to contact the guest editor (R.E.K.) to discuss a planned study, to learn if it would be considered suitable for publication, and if not, how to make it so.

## Data Availability

No human or animal data were used for this article.
